# Health‐related quality of life at 3 months following head and neck cancer treatment is a key predictor of longer‐term outcome and of benefit from using the patient concerns inventory

**DOI:** 10.1002/cam4.4558

**Published:** 2022-02-17

**Authors:** Anastasios Kanatas, Derek Lowe, Simon N. Rogers

**Affiliations:** ^1^ Leeds Teaching Hospitals and St James Institute of Oncology Leeds Dental Institute and Leeds General Infirmary Leeds UK; ^2^ Medical Statistician Astraglobe Ltd Congleton Cheshire UK; ^3^ Faculty of Health and Social Care Edge Hill University Ormskirk UK; ^4^ Liverpool Head and Neck Centre Liverpool University Hospital Aintree Liverpool UK

**Keywords:** health‐related quality of life, patient concerns inventory, head and neck cancer

## Abstract

**Introduction:**

During clinical follow‐up it can be difficult to identify those head and neck cancer (HNC) patients who are coping poorly and could benefit from additional support. Health‐related quality of life (HRQOL) questionnaires and prompt lists provide a means by which patients can express their perceived outcomes and raise concerns. The first aim of this secondary analysis following a randomized trial was to explore which patient characteristics, at around 3 months following treatment completion (baseline), best predict HRQOL 12 months later. The second aim was to attempt to ascertain which patients were most likely to benefit from using prompt list.

**Methods:**

Cluster‐controlled pragmatic trial data were analyzed. HRQOL was measured by the University of Washington Quality of life questionnaire (UW‐QOLv4). The prompt list was the Patient Concerns Inventory (PCI‐HN).

**Results:**

The trial involved 15 eligible consultants and a median (inter‐quartile range) of 16 (13–26) primary HNC patients per consultant, with 140 PCI patients and 148 controls. Baseline HRQOL was the dominant predictor of 12‐month HRQOL with other predictors related to social, financial, and lifestyle characteristics as well as clinical stage and treatment. Although formal statistical tests for interaction were non‐significant the trend in analyses over a range of outcomes suggested that patients with worse baseline HRQOL could benefit more from the PCI‐HN.

**Discussion:**

HRQOL early post‐treatment is a key predictor of longer‐term outcome. Measuring and using HRQOL and the PCI‐HN are not only surrogates for predicting HRQOL at 15 months post‐treatment, but also tools to help guide interventions.

## Graphical abstract

Health‐related quality of life (HRQOL) early post‐treatment is a key predictor of longer‐term outcome. Measuring and using HRQOL and the PCI‐HN are not only surrogates for predicting HRQOL at 15 months post‐treatment, but also tools to help guide interventions.

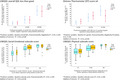

## 1 INTRODUCTION

Health‐related quality of life (HRQOL) is an established key outcome after treating patients for head and neck cancer (HNC), not only in the short term, but also longterm.^1^ There is now more understanding of the cancer journey long after treatment completion and we are starting to appreciate the variations at the individual‐patient level and their effect on HRQOL.

The Patient Concerns Inventory (PCI‐HN) prompt list is specific to HNC,^2^ that was designed to fit into routine clinic consultations. It is freely available and consists of 56 clinical items which patients select from prior to their appointment, to help guide their outpatient consultation through the symptoms and problems that they experience following treatment for HNC. It helps to direct the consultation and can trigger targeted onward referral for clinical areas of need and helps signpost patients for any advice and support they may need. It has been implemented in clinical care over the last 14 years and has undergone extensive development and validation. A systematic review of 14 self‐report measures recommended the use of the PCI‐HN to measure unmet needs, regarding content validity as being more important than quantitative psychometric properties.^3^ International data from 19 units provided an opportunity to reflect and supports the PCI approach in populations with different characteristics.^4^ The PCI‐HN prompt list allows HNC patients to discuss issues that might otherwise be overlooked.

A recent intervention trial evaluated the use of the PCI‐HN at routine outpatient clinics for 1 year after treatment on HRQOL.^5^ This trial integrated the PCI‐HN and the University of Washington Quality of life questionnaire (UW‐QOLv4) into routine consultations as a simple low‐cost means to benefit HNC patients. The UW‐QOL questionnaire is well established and has often been used in the last 20 years in HNC patients at different times after primary diagnosis. The UW‐QOLv4 and PCI‐HN can be used together in digital format in routine clinical practice with algorithms that can quickly identify patients doing badly, thus facilitating an immediate intervention from the clinical team.^6^ Published work indicated relationships between number of symptoms, functional status, physical status, and overall HRQOL.^7^ Previous work suggested that the number of PCI‐HN items were associated with overall HRQOL more strongly than with case‐mix variables^4^ of age, gender, tumor stage, site, and treatment.

There are many available studies looking at longitudinal changes in QOL‐ or trying to determine factors predictive of poor long‐term QOL‐, from pretreatment to 1 year, cross‐sectional to 5 years, even 10 years with a diverse use of instruments (EORTC, UW‐WOL).^1^, ^7^, ^8^, ^9^ With the introduction of the PCI in this study clinicians can evaluate patient needs in a longitudinal design, rather than looking at absolute scores.

We have reported previously^9^ that the HRQOL changes from 2 years to 10 years are minimal. There is flattening regarding change after 6 months‐to a year, that continues in long‐term survivorship.^9^ Regarding the population of patients included in this study we are not aware of any other randomized trials comparing HRQOL at different points within 2 years of diagnosis. Data from our recent trial^5^, ^10^ gave us the opportunity to look at the relevance of baseline patient baseline characteristics and their effect on HRQOL.

The first aim was to explore which baseline patient characteristics best predicted HRQOL outcome 12 months after being in the trial. The second aim was to explore which types of patients responded best to being in the PCI intervention arm of the trial and after completing the PCI prompt list at routine clinics throughout their trial follow‐up.

## 2 METHODS

A pragmatic cluster‐controlled trial was conducted at Aintree and Leeds cancer centres in the United Kingdom. Fifteen consultants (trial clusters) were randomized to “using” or “not using” the PCI prompt list intervention at all their routine outpatient clinics within the trial. The methods were detailed previously.^11^ Eligible patients were treated with curative intent for primary HNC, including all sites, stages, and treatments. Patients treated with palliative intent or those with recurrence were excluded as were patients with psychoses, cognitive impairment, or dementia. There was no limitation by age or histology. The PCI lists 56 clinical items^2^ and patients can select from these just before seeing their consultant, to help steer the outpatient consultation through a range of issues experienced after their treatment for HNC. MDT discussions about trial patients first took place meetings between January 2017 and December 2018, and the trial baseline clinics were from April 2017 to October 2019.

A baseline clinic questionnaire collected information as to whether patients lived alone or with others, whether they were working, had ever been unemployed, lived in a household receiving financial benefits, and the total household income before tax. Lifestyle factors about tobacco and alcohol use were also collected, as was patient ethnicity, gender, and age. Clinical details about primary tumor site, grade, treatment, and ACE27 comorbidity were obtained from clinical records. Index of multiple deprivation (IMD 2019) ranks were derived from patient postcodes using publicly available data and were analyzed as IMD quintile categories ranging from patients living in the 20% of most deprived small areas in England to patients in the 20% least deprived.

The UW‐QOLv4 contains 12 single question domains, with 3–5 evenly scaled responses scored from 0 (worst) to 100 (best).^12^ Regarding overall QOL, patients were asked to consider not only mental and physical health, but also other factors, such as family, friends, spirituality, or personal leisure activities important to their enjoyment of life. Subscale composite scores have been developed^13^ as have domain algorithms to screen for significant problems or dysfunction.^14^ Intimacy and fears of recurrence domain questions have also been developed using a similar concept of response hierarchy.^15^, ^16^ HRQOL data also included the Distress Thermometer (DT),^17^ where a score of 4 or greater reflects moderate to severe distress.

### 2.1 Statistical methods

The prespecified primary outcome measure of the trial was the percentage with less than good overall QOL (UWQOLv4) at 12 months. Two prespecified secondary outcomes at 12 months were the percentage with a DT score ≥4 and the mean social–emotional subscale score of the UWQOLv4. Other outcomes analyzed at 12 months were the mean physical functioning subscale of the UWQOLv4 and also whether there was dysfunction indicated specifically in each of the 12 UWQOL domains. HRQOL status at baseline clinic was analyzed as a predictor of status at 12 months alongside other baseline patient characteristics. The Mann–Whitney (2 comparison groups) or Kruskal–Wallis (>2 comparison groups) tests were used to compare patient groups for numerical outcomes and Fishers exact test was used for binary outcomes. Tests for interaction between baseline predictors and trial arm on 12‐month outcome were performed using logistic regression (binary outcomes) or linear regression (numerical outcomes). *R* ^2^ statistics estimating how much variation in an outcome was explained by a predictor variable, were obtained either from logistic regression (Nagelkerke pseudo *R* ^2^) for binary outcomes or from linear regression for numerical outcomes. The distribution of the number of UWQOL domains indicating dysfunction at 12 months (range 0–14) was skewed and regression methods incorporating bootstrapping methods (5000 replications) were used to estimate the significance of trial arm after adjusting for the baseline number of domain dysfunctions. Stepwise regression methods were used to select predictors of each outcome, and these allowed predicted probabilities (of binary outcomes, logistic regression) or expected scores (of numerical outcomes, linear regression) to be used to estimate the significance of trial arm after adjustment for the predictors. SPSSv25 and Statav13 were used for data analysis. In these exploratory analyses no allowance was made for clustering effects of the 15 consultants. Also, many statistical tests were performed and in general the more inferences being made, the more likely that erroneous inferences will occur. However, in the spirit of being exploratory and with the intent to be as inclusive of trends as possible we have retained our significance criteria at *p* < 0.05. Exploratory findings require confirmation by others.

## 3 RESULTS

There were 15 trial consultants and they saw a median (inter‐quartile range [IQR]) of 16 (13–26) patients, with 140 PCI‐HN patients and 148 controls. Patient flow charts from MDT to trial baseline clinic^10^ and from baseline to final trial clinic^5^ have been published. Baseline clinics took place a median (IQR) of 194 (125–249) days after diagnosis and 103 (71–162) days after patients ended their treatment. Baseline characteristics have been described.^5^, ^10^

Final clinic data were available for 71% in each group (PCI 100/140, non‐PCI 105/148), with 46% (38/83) lost due to cancer recurrence, palliation, 2nd primary, and death (PCI 45%, 18/40; non‐PCI 47%, 20/43) and 27% (22/83) due to early trial closure because of the COVID‐19 pandemic (PCI 28%,11/40; non‐PCI 26%, 11/43).

Final trial clinics (referred to as being at 12 months) for 100 PCI‐HN patients were a median (IQR) of 357 (329–380) days after trial baseline clinics, 364 (322–396) days for 105 controls. They were also a median (IQR) of 15.5 (13.8–17.2) months after the end of treatment. The median (IQR) number of PCI‐HN items selected were five (2–9) at the baseline clinic and two (0–4) at 12 months. The two trial groups were broadly similar in demographic and clinical characteristics apart from differences in tumor location and mode of treatment (Table 1) which were cluster (consultant) related with MFU and ENT consultants seeing different types of cases.

**TABLE 1:** Baseline demographic, lifestyle and clinical characteristics

	All patients		PCI patients		Non‐PCI patients	
	No.	%	No.	%	No.	%
Total	205	100	100	100	105	100
Location						
Aintree	119	58	55	55	64	61
Leeds	86	42	45	45	41	39
Age						
<55	49	24	20	20	29	28
55–64	87	42	45	45	42	40
65–74	46	22	23	23	23	22
≥75	23	11	12	12	11	10
Gender						
Male	143	70	65	65	78	74
Female	62	30	35	35	27	26
Tumour site						
Oral cavity	91	44	38	38	53	50
Oropharynx	70	34	33	33	37	35
Larynx	25	12	17	17	8	8
Other	19	9	12	12	7	7
Overall stage						
Early 0–2	89	43	40	40	49	47
Advanced 3–4	116	57	60	60	56	53
Treatment						
S only, no FF	73	36	36	36	37	35
S only, & FF	17	8	5	5	12	11
RT/CT only	39	19	26	26	13	12
S & RT/CT, no FF	51	25	24	24	27	26
S & RT/CT, & FF	25	12	9	9	16	15
ACE27 comorbidity						
None	107	52	58	58	49	47
Mild	61	30	26	26	35	33
Mod/severe	37	18	16	16	21	20
Ethnic group						
White British	198	97	98	98	100	95
Other	7	3	2	2	5	5
IMD 2019 (quintile)						
1 worst	72	35	35	35	37	35
2	22	11	12	12	10	10
3	40	20	18	18	22	21
4	45	22	23	23	22	21
5 best	26	13	12	12	14	13
Currently living in house or flat						
With other	165	81	82	82	83	80
Alone	39	19	18	18	21	20
Not known	1				1	
Currently working						
Yes	72	36	41	43	31	30
No	128	64	55	57	73	70
Not known	5		4		1	
Ever been unemployed						
Yes	69	35	32	34	37	36
No	129	65	63	66	66	64
Not known	7		5		2	
Household receives financial benefits						
None	125	65	64	68	61	62
Yes	68	35	30	32	38	38
Not known	12		6		6	
Total household income from all sources before tax						
<£12,000	35	17	14	14	21	20
£12,000–22,999	31	15	16	16	15	14
£23,000–34,999	37	18	19	19	18	17
≥£35,000	46	22	21	21	25	24
Not known	56	27	30	30	26	25
Tobacco user						
Current	24	12	12	12	12	12
Former	117	59	60	61	57	56
Never	59	30	26	27	33	32
Not known	5		2		3	
Alcohol user						
Current	146	73	80	81	66	65
Former	45	22	15	15	30	29
Never	10	5	4	4	6	6
Not known	4		1		3	

Abbreviations: CT, chemotherapy; FF, free‐flap; RT, radiotherapy; S, surgery.

**FIGURE 1:**
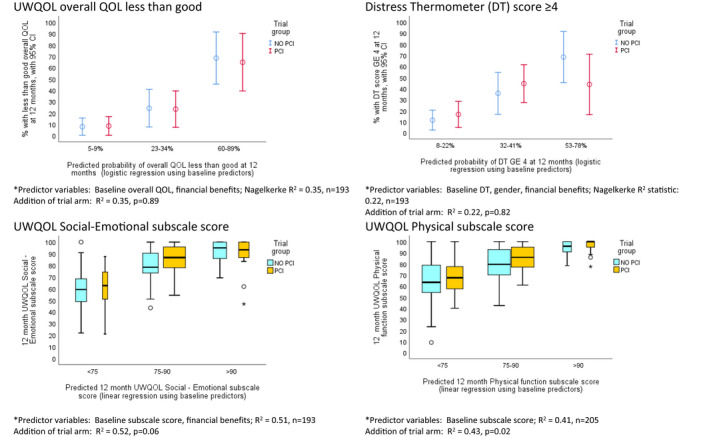
Main trial HRQOL outcomes at 12 months, by predictions of HRQOL and trial arm. *From stepwise logistic regression (overall QOL & DT) and linear regression (subscale scores) analyses of outcome on the predictor variables of Table 1 (omitting unknowns apart from income). For the subscale scores an asterisk * represents a value more than 3 box lengths from the upper or lower edge of the box, while a circle O marks a value between 1.5 and 3 box lengths away from the box. 95% CI, approximate 95% confidence interval; DT, Distress Thermometer; HRQOL, health‐related quality of life

At 12 months, 23% (48/205) reported less than good overall (UWQOL) quality of life, 32% (65/205) a DT score of 4 or more, social–emotional median (IQR) subscale scores of 87 (71–96), mean 81, and physical function median (IQR) subscale scores of 80 (66–95), mean 78. Significant associations of baseline casemix characteristics (Table 1) with main trial outcomes are shown in Table 2, with results stratified by trial arm. Baseline HRQOL was the dominant predictor of 12‐month HRQOL with PCI patients tending to have better outcomes than controls when baseline HRQOL was worst. Formal tests for interaction between trial arm and baseline HRQOL on 12‐month outcomes were all non‐significant. Worse outcomes were noted for those in households receiving benefits, currently not working, or with household incomes under £12,000. Clinical stage and treatment associated with physical function scores. Across Table 2 there were no significant interactions between baseline predictors and trial arm on outcome apart from patients ever having been unemployed (yes/No) in regard to less than good overall QOL (*p* = 0.03). Table 2 predictors were entered into stepwise regression models (*p* < 0.05 entry) and from final models predicted probabilities (of overall QOL less than good and DT score ≥ 4) and predicted scores (of UWQOL subscale scores) were obtained for each patient. These 12‐month outcome predictions were plotted against actual outcome for each arm of the trial (Figure 1) to assess the trial arm effect after adjusting for the regression predictors. After such adjustment, PCI‐HN patients tended to have better outcome results at 12 months for the UWQOL subscale outcomes while differences regarding overall QOL were weaker and inconsistent for DT.

**TABLE 2:** Notable associations (*p* < 0.05) between baseline variables and key HRQOL outcomes at 12 months, with results stratified by trial arm

12 month outcome	Baseline predictor variable	Trial arm	Observed nature of association, with 12 month outcomes reported				
Overall QOL less than good: % (*n*)	IMD 2019		Worst 2 quintiles^a^	Best 3 quintiles			
	*p* = 0.01	PCI	26% (12/47)	19% (10/53)			
	*R* ^2^ = 0.05	No PCI	38% (18/47)	14% (8/58)			
	Currently working		Working	Not working			
	*p* = 0.002	PCI	7% (3/41)	35% (19/55)			
	*R* ^2^ = 0.08	No PCI	16% (5/31)	29% (21/73)			
	Ever unemployed		Yes	No			
	*p* = 0.04	PCI	22% (7/32)	24% (15/63)			
	*R* ^2^ = 0.03	No PCI	43% (16/37)	15% (10/66)			
	Financial benefits		Benefits	No benefits			
	*p* < 0.001	PCI	40% (12/30)	16% (10/64)			
	*R* ^2^ = 0.17	No PCI	47% (18/38)	10% (6/61)			
	Household income		<£12,000	£12,000–22,999	£23,000–34,999	≥£35,000	Not known
	*p* = 0.002	PCI	57% (8/14)	19% (3/16)	16% (3/19)	19% (4/21)	13% (4/30)
	*R* ^2^ = 0.12	No PCI	48% (10/21)	13% (2/15)	11% (2/18)	16% (4/25)	31% (8/26)
	Overall QOL		Poor/very poor	Fair	Good	Very good/outstanding	
	*p* < 0.001	PCI	67% (6/9)	38% (8/21)	12% (4/34)	11% (4/36)	
	*R* ^2^ = 0.23	No PCI	83% (5/6)	46% (10/22)	18% (6/33)	11% (5/44)	
DT score ≥4: % (*n*)	Gender		Male	Female			
	*p* = 0.02	PCI	28% (18/65)	43% (15/35)			
	*R* ^2^ = 0.04	No PCI	26% (20/78)	44% (12/27)			
	Tumour site		Oral cavity	Oropharynx	Larynx	Other	
	*p* = 0.04	PCI	42% (16/38)	33% (11/33)	24% (4/17)	17% (2/12)	
	*R* ^2^ = 0.06	No PCI	38% (20/53)	30% (11/37)	13% (1/8)	0% (0/7)	
	IMD 2019		Worst 2 quintiles^a^	Best 3 quintiles			
	*p* = 0.02	PCI	36% (17/47)	30% (16/53)			
	*R* ^2^ = 0.04	No PCI	45% (21/47)	19% (11/58)			
	Financial benefits		Benefits	No benefits			
	*p* = 0.003	PCI	40% (12/30)	28% (18/64)			
	*R* ^2^ = 0.06	No PCI	47% (18/38)	18% (11/61)			
	Household income		<£12,000	£12,000–22,999	£23,000–34,999	≥£35,000	Not known
	*p* = 0.03	PCI	36% (5/14)	25% (4/16)	26% (5/19)	33% (7/21)	40% (12/30)
	*R* ^2^ = 0.07	No PCI	57% (12/21)	20% (3/15)	11% (2/18)	20% (5/25)	38% (10/26)
	DT score (0–10)		Zero	1–3	4–5	6–10	
	*p* < 0.001	PCI	18% (5/28)	37% (10/27)	36% (8/22)	43% (10/23)	
	*R* ^2^ = 0.14	No PCI	12% (4/33)	21% (7/34)	38% (6/16)	68% (15/22)	
UWQOL social‐emotional subscale score: median (IQR), mean, *n*	Currently working		Working	Not working			
	*p* = 0.006	PCI	90 (80–98), 86.8, *n* = 41	87 (73–96), 81.8, *n* = 55			
	*R* ^2^ = 0.05	No PCI	86 (72–96), 84.3, *n* = 31	78 (62–91), 74.8, *n* = 73			
	Financial benefits		Benefits	No benefits			
	*p* < 0.001	PCI	78 (70–91), 78.5, *n* = 30	90 (79–96), 86.4, *n* = 64			
	*R* ^2^ = 0.14	No PCI	67 (51–84), 67.6, *n* = 38	87 (73–95), 83.8, *n* = 61			
	Household income		<£12,000	£12,000–22,999	£23,000–34,999	≥£35,000	Not known
	*p* = 0.002	PCI	73 (53–82), 70.9, *n* = 14	93 (75–100), 87.9, *n* = 16	88 (83–95), 85.4, *n* = 19	87 (78–100), 85.1, *n* = 21	88 (78–96), 86, *n* = 30
	*R* ^2^ = 0.11	No PCI	71 (57–88), 69.7, *n* = 21	83 (74–95), 80.8, *n* = 15	76 (70–96), 78.3, *n* = 18	91 (76–96), 84.0, *n* = 25	78 (65–91), 76, *n* = 26
	Alcohol use		Current	Former	Never		
	*p* = 0.005	PCI	88 (78–96), 85.1, *n* = 80	74 (67–92), 77.6, *n* = 15	95 (na), 85.4, *n* = 4		
	*R* ^2^ = 0.06	No PCI	83 (71–95), 79.8, *n* = 66	72 (61–87), 72.5, *n* = 30	88 (na), 80.8, *n* = 6		
	UWQOL Social‐emotional subscale score quintile^b^		≤55.8	55.9–70.0	70.1–81.7	81.8–90.8	≥90.9
		PCI	65 (45–74), 58.8, *n* = 10	83 (69–87), 78.2, *n* = 21	91 (77–98), 87.7, *n* = 25	91 (87–97), 89.8, *n* = 18	94 (87–100), 90.3, *n* = 26
	*p* < 0.001 *R* ^2^ = 0.46	No PCI	57 (46–70), 53.7, *n* = 19	73 (63–84), 72.0, *n* = 22	78 (71–90), 79.0, *n* = 20	87 (76–95), 85.7, *n* = 16	96 (88–100), 93.4, *n* = 28
UWQOL physical function subscale score: median (IQR), mean, *n*	Overall stage		Early 0–2	Advanced 3–4			
	*p* = 0.002	PCI	90 (78–96), 87.3, *n* = 40	78 (65–91), 77.4, *n* = 60			
	*R* ^2^ = 0.07	No PCI	86 (68–96), 81.1, *n* = 49	73 (60–83), 70.9, *n* = 56			
	Treatment		S only no FF	S only & FF	RT/CT only	S & RT/CT no FF	S & RT/CT & FF
	*p* < 0.001	PCI	93 (79–100), 89.4, *n* = 36	71 (na), 75.5, *n* = 5	74 (65–88), 76.1, *n* = 26	85 (69–91), 81.0, *n* = 24	75 (na), 68.0, *n* = 9
	*R* ^2^ = 0.16	No PCI	90 (73–100), 83.6, *n* = 37	83 (67–94), 78.5, *n* = 12	74 (50–80), 65.1, *n* = 13	78 (66–95), 75.7, *n* = 27	63 (51–78), 63.8, *n* = 16
	Financial benefits		Benefits	No benefits			
	*p* = 0.005	PCI	78 (66–95), 78.8, *n* = 30	85 (73–95), 82.9, *n* = 64			
	*R* ^2^ = 0.09	No PCI	67 (58–79), 67.0, *n* = 38	86 (70–95), 80.7, *n* = 61			
	Household income		<£12,000	£12,000–22,999	£23,000–34,999	≥£35,000	Not known
	*p* = 0.03	PCI	77 (55–95), 74.5, *n* = 14	82 (72–94), 82.6, *n* = 16	83 (72–96), 81.5, *n* = 19	82 (71–96), 81.2, *n* = 21	87 (74–95), 84, *n* = 30
	*R* ^2^ = 0.07	No PCI	66 (58–78), 67.3, *n* = 21	79 (61–100), 79.3, *n* = 15	88 (70–95), 79.4, *n* = 18	83 (70–95), 80.4, *n* = 25	78 (56–91), 73, *n* = 26
	UWQOL physical function score (quintiles)^b^		≤50.00	50.01–65.00	65.01–72.50	72.51–90.00	≥90.01
		PCI	61 (54–70), 62.4, *n* = 12	74 (59–83), 71.8, *n* = 23	82 (73–90), 81.5, *n* = 21	90 (81–95), 88.7, *n* = 27	100 (93–100), 95.5, *n* = 17
	*p* < 0.001 *R* ^2^ = 0.41	No PCI	58 (38–73), 56.5, *n* = 19	68 (57–80), 68.9, *n* = 20	74 (67–86), 74.5, *n* = 25	88 (74–95), 84.4, *n* = 23	96 (90–100), 93.8, *n* = 18

*Note*: The baseline predictor variable *p* values (2nd column) came from Fishers Exact test in regard to overall QOL and DT outcomes, or from Mann‐Whitney (2 group comparison) or Kruskal‐Wallis (>2 groups) tests regarding the subscale score outcomes. The *R* ^2^ statistics (also 2nd column) were derived from logistic regression (Nagelkerke pseudo *R* ^2^) in regard to overall QOL and DT and from linear regression for the subscale scores

Abbreviations: CT, chemotherapy; na, IQR was not computed for denominators <10; RT, radiotherapy; S, surgery.

^a^ IMD 2019: ‘Worst 2 quintiles’ indicates those patients living in the most deprived 40% of English small area neighbourhoods; ‘Best 3 quintiles’ indicate patients living in other less deprived small area neighbourhoods.

^b^ Quintiles derived from baseline sample of 288.

**FIGURE 2:**
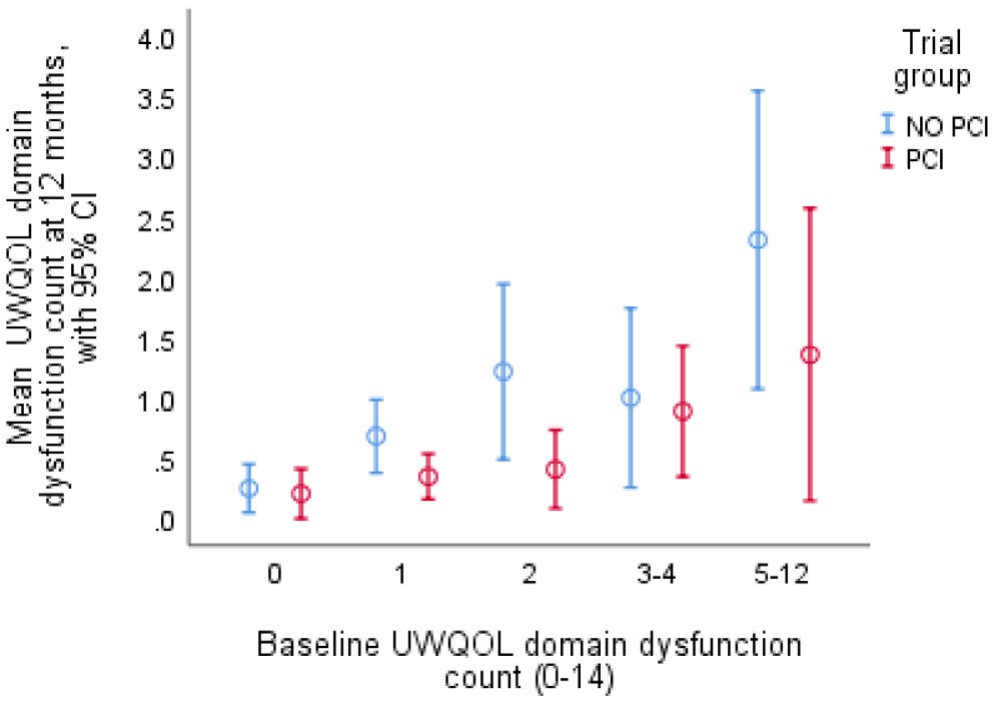
Number of 12‐month UWQOL dysfunction outcomes by baseline status and trial arm. From linear regression analysis using bootstrapping methods (5000 replications) the PCI effect was significant (*p* = 0.013) on the number of dysfunctional domains per patient at 12 months after adjusting for baseline number of dysfunctional domains (0, 1, 2, 3–4, 5–12). 95% CI: approximate 95% confidence interval number of patients at baseline with: 0 dysfunctional domains (27 PCI, 30 no PCI), 1 dysfunction (33 PCI, 33 no PCI), 2 dysfunctions (18 PCI, 17 no PCI), 3–4 dysfunctions (15 PCI, 12 no PCI), and 5–12 dysfunctions (7 PCI, 13 no PCI). PCI, Patient Concerns Inventory; UW‐QOLv4, University of Washington Quality of life questionnaire

UWQOL domain dysfunction at 12 months ranged from 22% (saliva), 13% (anxiety), and 12% (pain) to 3% (recreation and speech) and 2% (appearance and intimacy). Nearly half (48%, 98/205) had at least one domain dysfunction while 24% (49/205) had two or more dysfunctions and 9% (19/205) had four or more. When results for the number of domain dysfunctions at 12 months were stratified by the number of domain dysfunctions at baseline the tendency was for PCI‐HN patients on average to have less 12‐month dysfunction than control patients (Figure 2), the independent effect of PCI‐HN being *p* = 0.01 after adjusting for baseline number of dysfunctions. Regarding each of the 14 specific domains the baseline domain status (dysfunction, best possible score, in‐between status) was the dominant baseline predictor of 12‐month dysfunction (Table 3). The tendency across domains was for PCI‐HN patients to have less 12‐month dysfunction than controls when baseline status was sub‐optimal (dysfunction or intermediate) while outcomes were more similar for those with the best possible domain baseline scores (Results not shown). Stepwise logistic regressions (*p* < 0.05 for entry) of 12‐month dysfunction for each domain always selected baseline domain status as the first predictor and additional predictors were selected for anxiety (+benefits), speech (+ever unemployed), taste (+tumor stage), and saliva (+benefits). For each situation the extra independent effect of PCI‐HN was assessed and this was significant for swallowing (*p* = 0.01) and chewing (*p* = 0.04).

**TABLE 3:** Notable associations (*p* < 0.05) between baseline status and UWQOL dysfunction at 12 months

12 month UWQOL outcome	Overall result (*n* = 205)	Baseline status and 12 month outcome result: % (*n*)			
		Baseline status	Best baseline score	In‐between baseline status	Baseline dysfunction
Dysfunction in UWQOL social‐emotional subscale domains					
Pain	12% (24)	*p* = 0.001, *R* ^2^ = 0.14	4% (3/80)	10% (7/71)	26% (14/54)
Activity	6% (12)	*p* < 0.001, *R* ^2^ = 0.25	2% (1/65)	3% (4/119)	33% (7/21)
Recreation	3% (7)	*p* < 0.001, *R* ^2^ = 0.36	1% (1/89)	1% (1/101)	33% (5/15)
Shoulder	6% (13)	*p* = 0.001, *R* ^2^ = 0.16	2% (2/119)	10% (6/61)	20% (5/25)
Mood	9% (18)	*p* < 0.001, *R* ^2^ = 0.32	1% (1/75)	6% (6/106)	46% (11/24)
Anxiety	13% (27)	*p* < 0.001, *R* ^2^ = 0.13	6% (5/83)	12% (11/92)	37% (11/30)
Dysfunction in UWQOL physical function subscale domains					
Appearance	2% (5)	*p* = 0.002, *R* ^2^ = 0.24	2% (1/55)	1% (1/136)	21% (3/14)
Swallowing	6% (12)	*p* < 0.001, *R* ^2^ = 0.33	1% (1/78)	3% (3/106)	38% (8/21)
Chewing	5% (10)	*p* < 0.001, *R* ^2^ = 0.27	1% (1/84)	3% (3/103)	33% (6/18)
Speech	3% (7)	*p* < 0.001, *R* ^2^ = 0.63	0% (0/90)	1% (1/105)	60% (6/10)
Taste	9% (19)	*p* < 0.001, *R* ^2^ = 0.32	3% (2/68)	3% (3/100)	38% (14/37)
Saliva	22% (46)	*p* < 0.001, *R* ^2^ = 0.13	8% (5/59)	18% (14/77)	39% (27/69)
Dysfunction in extra UWQOL domains					
Intimacy	2% (5)	*p* < 0.001, *R* ^2^ = 0.37	0% (0/158)	8% (3/37)	20% (2/10)
Fears of recurrence	6% (13)	*p* = 0.002, *R* ^2^ = 0.28	0% (0/36)	5% (8/152)	29% (5/17)

*Note*: *p* values came from Fishers Exact test while the *R* ^2^ statistics (Nagelkerke pseudo *R* ^2^) were estimated from using logistic regression.

**FIGURE 3:**
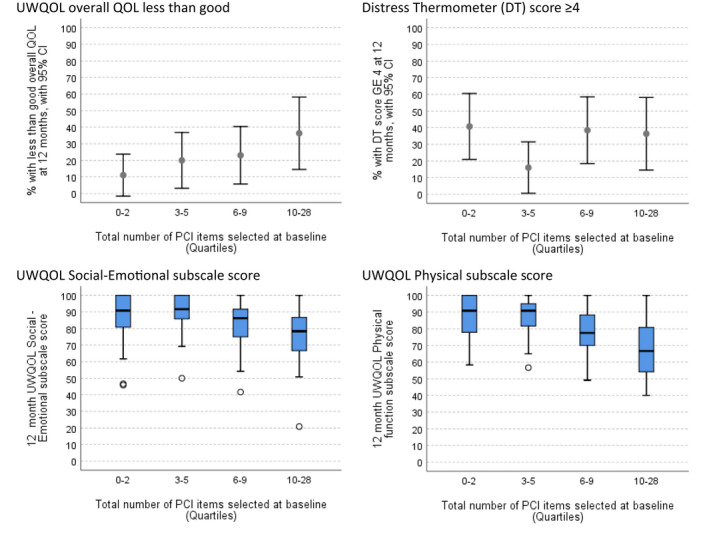
Main trial HRQOL outcomes at 12 months, by number of PCI items selected at baseline. For the subscale scores a circle O marks a value between 1.5 and 3 box lengths away from the box. 95% CI, approximate 95% confidence interval; HRQOL, health‐related quality of life; PCI, Patient Concerns Inventory

For PCI‐HN patients the more PCI‐HN items they selected at baseline the greater the number likely to be selected at 12 months: 52 patients selecting 0–5 items at baseline selected a median of 1 (mean 1.7) items at 12 months, 26 patients selecting 6–9 at baseline selected a median of 2 (mean 3.4) items at 12 months, while 22 patients selecting 10–28 items at baseline selected a median of 5 (mean 7.1) items at 12 months. Also, the more PCI‐HN items selected at baseline the greater the percentage of patients at 12 months having less than good overall QOL and the worse the scores for the two UWQOL subscales (Figure 3); no trend was seen for DT scores of 4 and above.

## 4 DISCUSSION

A strength of this study is the details collected at baseline and the prospective collection of HRQOL and PCI‐HN data. To our knowledge this is the only randomized trial that evaluates the HRQOL at 3 months (post treatment end) and 12 months later within the stated patient group. This paper highlights patient characteristics that predict HRQOL outcomes at around 15 months following treatment. Prominent among these, is the starting (posttreatment) HRQOL level.

Previous retrospective cohort studies have suggested that baseline (pre‐treatment) HRQOL strongly influences posttreatment HRQOL with greater impact than treatment modality.^18^ The importance of early HRQOL measurement, just after treatment completion, is highlighted by our intervention trial. It is known from already published work that the patients' HRQOL deteriorates just after treatment and subsequently improves, slowly toward pretreatment scores, after 1 year.^19^ Furthermore, patient concerns decrease as these patients progressed in their recovery.^20^ The study by Aminnudin et al. (2020) revealed a significant association between the number of PCI‐HN items selected and the “time after treatment completed” (*p* < 0.001). They^20^ observed that a high number of concerns were strongly associated with patients in the “1‐month to 1‐year post‐treatment”. The same study also suggested an association between the number of concerns and the patients' HRQOL, and although they did not specifically look at the HRQOL at 3 months (posttreatment end) and 12 months later, their results are indirectly supporting the data presented in this paper.

Recommendations regarding the frequency of HRQOL measurement have been published as early as 2003 and may include collection of data at multiple points in the cancer journey.^21^ The vast majority of oncological studies that report on HRQOL include measurements during or shortly after treatment.^22^ Recommendations include the frequent use of different outcome measures for evaluating patient well‐being.^23^ In an ideal setting policy recommendation and the evaluation of different treatment modalities on HRQOL, should be based on QOL outcomes throughout the patients' cancer care; however, taking into account the available resources this may not be possible. The analyses suggested that the PCI‐HN intervention impacts on the social emotional and physical function subscales, and this also showed through in the analyses of domain dysfunction. The evidence regarding overall HRQOL and DT appears weak, as already inferred from the primary outcomes paper. The trial effect on the social emotional and physical function subscale scores at 12 months seems small in absolute terms when compared to the relationship noted for other predictors, especially baseline HRQOL––but this is often the case with randomized trial effects––a series of small gains. Although tabulated results might suggest variation in how patients in different baseline subgroups respond to using the PCI, there was little formal statistical test evidence of interaction. The resulting logic is that any observed variation in results regarding the trial effect in different patient subgroups is consistent with chance/random variation.

### 4.1 Study limitations

The trial ended up being underpowered generally, partly because of early termination due to the COVID‐12 pandemic but also the greater than expected loss in the time lag between the multidisciplinary team meeting and trial posttreatment baseline clinics. The analyses were deliberately exploratory, the prespecified main analyses having already been reported. The intention was to be inclusive of trends and impressions rather than be parsimonious, but in so doing it is accepted that the more inferences that are made the more likely that erroneous inferences will occur. By definition exploratory findings require confirmatory analyses from other researchers. However, the results do offer more clarity as to predictors of 12‐month outcome than about which groups of patients benefit most from using the PCI‐HN. Baseline HRQOL status looks to be the dominant predictor of 12‐month outcomes and the trend in analyses over a range of outcomes suggests that patients with worse baseline HRQOL could benefit more from the PCI‐HN. The findings from this work are applicable to any population with similar ethnic and socioeconomic characteristics.

### 4.2 Clinical implications

The explanation for why 3 month posttreatment scores may be predictive of 1 year scores need further research. Many factors will contribute to this and not only include treatment effects, but also included other psychosocial factors such as resilience, coping mechanisms, personality, as well as the interactions between patients and family members or carers. Further explanatory and intervention studies are required to explain how the PCI prompt list aids adaptation following HNC.

From the results of this work the starting HRQOL (just after treatment) measurement with the concurrent use of the PCI‐HN, could be the foundation for treatment assessment and target early interventions, with long‐term benefits. Other notable predictors were individual social, financial, and lifestyle factors as well as characteristics of the area in which patients lived; also, clinical stage and treatment predicted physical function. The financial burden of cancer was highlighted in a previous cross‐sectional study^24^ several years ago. Despite that, almost 10 years later this is still an issue that multidisciplinary teams need to do more about, by signposting early, appropriate available benefits for patients and carers. In conclusion, measuring the HRQOL early after the completion of treatment provides an indication of the likely HRQOL at 12 months later. By acting on both HRQOL scores and PCI‐HN concerns, clinicians can make a valuable contribution to improve outcomes for their patients. Further research is needed to develop suitable and effective interventions to improve the long‐term HRQOL.

## CONFLICT OF INTERESTS

The authors declare that they have no known competing financial interests or personal relationships that could have appeared to influence the work reported in this paper.

## AUTHOR CONTRIBUTIONS

This study could not have taken place without the valued contribution and support of the 15 consultants who participated in this trial. Anastasios Kanatas contributed to the design, delivery of trial and collection of data, and writing the manuscript, Derek Lowe contributed to the design and methodology of the trial, data analysis, and the writing of the manuscript and Simon N. Rogers contributed the design, delivery of trial and collection of data, and writing the manuscript. Simon N. Rogers was the chief investigator of the trial. The final manuscript was approved by all authors.

## ETHICAL APPROVAL STATEMENT

The study complied with all aspects of ethical standards of clinical research. Ethical approval from North West‐Liverpool Central Research Ethics Committee REC reference: IRAS 16/NW/0465, Project ID: 189554. The PCI trial has approval from the Health Research Authority (HRA). The Research and Development Department at Aintree University Hospital NHS Trust (AUH) is coordinating the trial and AUH is the sponsor for the trial. Trial registration: 32,382. Clinical Trials Identifier, NCT03086629.

## PATIENT CONSENT STATEMENT

Participants provided written informed consent. A patient information leaflet was provided and the patients had an opportunity to decide if they would like to take part in the trial, after a period of 2 weeks.

## Data Availability

The data that support the findings of this study are available from the corresponding author upon reasonable request.
